# Real-World Implications of Nonbiological Factors with Staging, Prognosis and Clinical Management in Colon Cancer

**DOI:** 10.3390/cancers10080263

**Published:** 2018-08-08

**Authors:** Qi Liu, Dakui Luo, Sanjun Cai, Qingguo Li, Xinxiang Li

**Affiliations:** 1 Department of Colorectal Surgery, Fudan University Shanghai Cancer Center, Shanghai 200032, China; LQ671993@163.com (Q.L.); 15262905600@163.com (D.L.); 16211230008@fudan.edu.cn (S.C.); 2 Department of Oncology, Shanghai Medical College, Fudan University, Shanghai 200032, China

**Keywords:** non-biological factors, AJCC TNM staging system, prognostication, colon cancer, SEER

## Abstract

*Background:* The present study analyzed the nonbiological factors (NBFs) together with the American Joint Committee on Cancer (AJCC) Tumor-Node-Metastasis (TNM) staging system to generate a refined, risk-adapted stage for the clinical treatment of colon cancer. *Methods:* Eligible patients (*N* = 28,818) with colon cancer between 1 January 2010 and 31 December 2014, were identified from the Surveillance, Epidemiology, and End Results (SEER) database. Kaplan-Meier curves and Cox proportional hazards regression, analyzed the probabilities of cancer-specific survival (CSS) in patients with colon cancer, with different NBF-TNM stages. *Results:* Insurance status, marital status, and median household income were significant prognostic NBFs in the current study (*p* < 0.05). The concordance index of NBF-TNM stage was 0.857 (95% confidence interval (CI) = 0.8472–0.8668). Multivariate Cox analyses, indicated that NBF1-stage was independently associated with a 50.4% increased risk of cancer-specific mortality in colon cancer (*p* < 0.001), which increased to 77.1% in non-metastatic colon cancer. NBF0-stage improved in CSS as compared to the NBF1-stage in the respective stages (*p* < 0.05). *Conclusions:* The new proposed NBF-stage was an independent prognostic factor in colon cancer. Effect of NBFs on the survival of colon cancer necessitates further clinical attention. Moreover, the incorporation of NBF-stage into the AJCC TNM staging system is essential for prognostic prediction, and clinical guidance of adjuvant chemotherapy in stage II and III colon cancer.

## 1. Introduction

Colon cancer is one of the most common malignancies and its increasing incidence has been noted over the years in the USA [1]. The prognosis of patients with malignant colon cancer is influenced both by disease factors and patient-related factors, including biological factors and nonbiological factors (NBFs). The effect of different biological factors, such as American Joint Committee on Cancer (AJCC) staging system, microsatellite instability (MSI) status, and tumor grade on the survival of patients with colon cancer has been widely studied [2,3,4]. Several studies have demonstrated that NBFs, such as marital status [5,6], health insurance [7,8], and employment [9] were associated with the survival of patients with colon cancer. However, to the best of our knowledge, NBFs have not yet been studied together in the prognosis of colon cancer. The prognostication of AJCC staging system is only based on the invasion extent of the primary tumor (T stage), lymph node status (N stage), and distant spread (M stage) [10]. It is not perfect for prognostic prediction and clinical management, and a better prognostic staging system combined with AJCC staging system and other prognostic factors is needed [10,11,12].

Herein, we conducted a large population-based study to analyze the effect of different NBFs, such as employment, education, income, health insurance, year of diagnosis, and marital status on survival in colon cancer cases. Thus, we proposed and evaluated a novel NBF-TNM stage (i.e., combination of AJCC staging system and NBF stage), with respect to the prediction of prognosis and clinical management.

## 2. Patients and Methods

Ethics Statement: This study was based on public data from the freely available SEER database and was approved by the Ethical Committee and Institutional Review Board of the Fudan University Shanghai Cancer Center. We obtained permission to access research data files with the reference number 10782-Nov2016 and the permission date was 8 April 2017. The data did not include the use of human subjects or personal identifying information and no informed consent was required for this study.

### 2.1. Study Design and Data Source

The Surveillance, Epidemiology, and End Results (SEER) database is an authoritative source of information on cancer incidence and survival in the USA. It is a comprehensive source of population-based information, including all the newly diagnosed cancer cases occurring in individuals residing in SEER-participating areas, encompassing approximately 28% of the American population.

**Figure 1:**
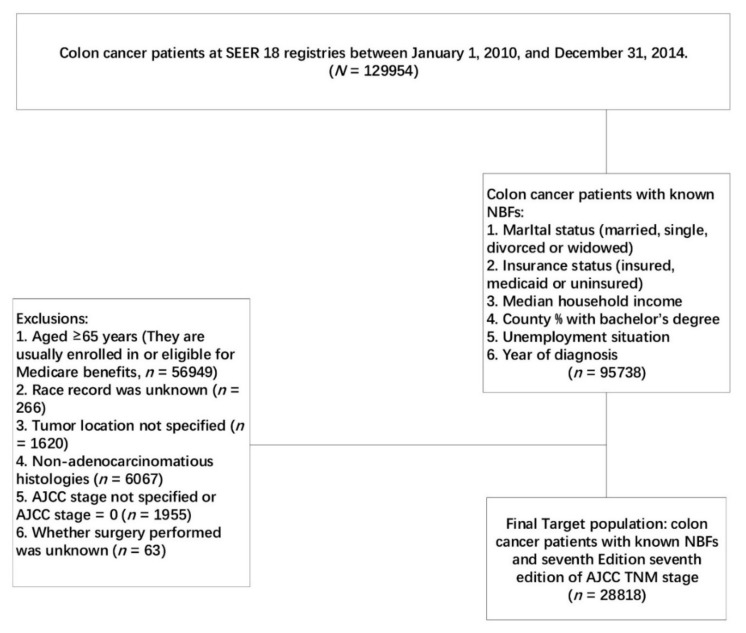
Flow diagram of patient population selected from Surveillance, Epidemiology, and End Results (SEER) database.

Using the SEER-Stat software (SEER*Stat 8.3.4, https://seer.cancer.gov/seerstat/software/), patients diagnosed with colon cancer between 1 January 2010 and 31 December 2014, from the SEER Program of the National Cancer Institute were identified, as shown in Figure 1.

Among these patients with colon cancer, those with known NBFs (including marital status, insurance status, county-level median household income, county percentage with a bachelor’s degree, unemployment situation, and year of diagnosis) were included in our analyses. Considering that the insurance status was included in our analyses as an NBF, patients whose age at diagnosis ≥65 years were excluded from the population, as most of them were eligible for Medicare benefits. Patients with unknown race, unspecified tumor location, non-adenocarcinomatous histology, unspecified seventh AJCC stage, and those with seventh AJCC stage = 0 or whether surgery performed was unknown, were also excluded from the current analyses.

### 2.2. NBF Stage and Statistical Analysis

Cox proportional hazards models were established to identify the independent prognostic variables at a median survival time of 21 (range, 0–59) months. The hazard ratios were shown with 95% confidence intervals (CIs). Thus, we conducted a multivariate Cox proportional hazard regression analysis of all the prognostic factors associated with *p*-value < 0.2 in the univariate analysis, including the NBFs (marital status, insurance status, county-level median household income, county percentage with a bachelor’s degree, unemployment situation, and year of diagnosis). The results showed that marital status, insurance status, and county-level median household income were significant prognostic NBFs of CSS in colon cancer.

**Figure 2:**
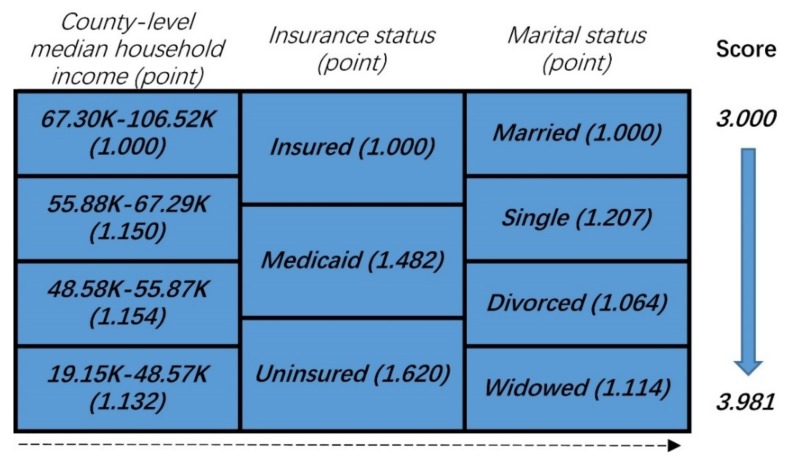
Patient prognostic score in colon cancer: risk-stratifications.

**Figure 3:**
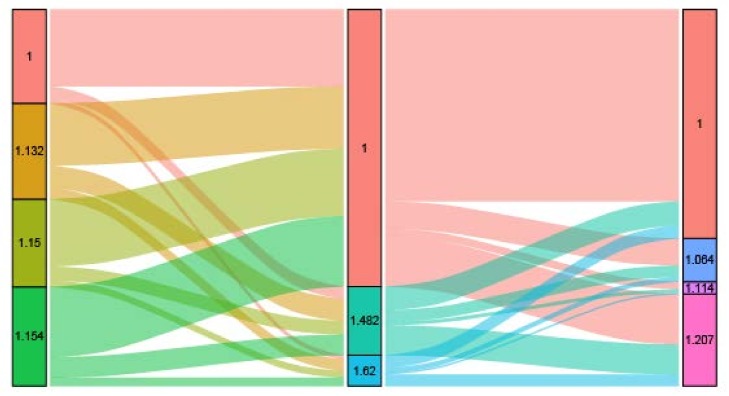
Graphical summary of the distribution and associations of different score subgroups in county-level median household income, insurance status and marital status, respectively.

As shown in Figure 2, patients were stratified based on the prognostic score incorporating the three NBFs. First, we considered the point of each group of NBFs equivalent, to the value of the hazard ratios. Then, the total prognostic score of each patient was calculated as the sum of the points in the three NBFs. For example, a married and uninsured colon cancer patient whose county-level median household income was 48.58–55.87 K (dollars), the score was calculated as the sum of “1.150”, “1.620”, and “1.000” which equaled to “3.770”. The total scores ranged from 3.000–3.981, followed by a comprehensive prognostic score based on the three NBFs, which was 3.000 with optimal prognosis and those with a score of 3.981 had the worst prognosis. The distribution and associations of different score subgroups, are shown in Figure 3. Finally, the prognostic score was divided into two groups, and the cut-off point was the median value of the prognostic score of the whole population. The higher score was assigned to stage NBF1, while the other was assigned to stage NBF0.

Multivariate Cox proportional hazard regression analysis determined the prognosis of NBF stage, and the combination of AJCC TNM staging system and NBF stage (TNM-N stage). The endpoint used for comparison in the present study was CSS. The Kaplan–Meier survival curves were used to evaluate the prognostic prediction of different factors and the log-rank tests, to assess the statistical significance. A *p*-value < 0.05 was considered statistically significant. The statistical analysis was performed using the Statistical Package for Social Science (SPSS version 22; IBM Corporation, Armonk, NY, USA).

## 3. Results

A total of 28,818 patients were diagnosed with colon cancer between 1 January 2010 and 31 December 2014, from the SEER Program. The median follow-up time was 21 (range, 0–59) months. At the end of the follow-up time, 4404 (15.3%) patients had died of colon cancer. The baseline characteristics of colon cancer patients included in the current study were summarized in Table 1.

### 3.1. Three NBFs Were Strongly Associated with CSS of Colon Cancer

Univariate analysis demonstrated that race, gender, tumor location, tumor grade, AJCC stage, surgery, insurance status, marital status, tumor size, age at diagnosis, county percentage with bachelor’s degree, county-level median household income, and county percentage of unemployed were associated with CSS (*p* < 0.2). These factors were included in the multivariate Cox hazard regression analysis, and the result showed that NBFs such as insurance status, marital status, and county-level median household income were independently associated with CSS (Table 2). Other factors identified as independent protective factors included race, gender, tumor location, tumor grade, AJCC stage, surgery, tumor size, and age at diagnosis.

### 3.2. NBF Stage Was Strongly Associated with CSS in Colon Cancer

NBF0-stage was assigned to 15,326 patients (53.2%) and NBF1-stage was assigned to 13,492 patients (46.8%). Multivariable analysis showed that the NBF1 was independently associated with CSS of 28,818 patients with colon cancer, with a 50.4% increased risk of cancer-specific mortality (hazard ratio (HR) = 1.504, 95% CI: 1.415–1.600, *p* < 0.001; Table 3). A multivariable Cox analysis was also conducted in patients with non-metastatic colon cancer (*n* = 22,149), on the overall cohort, which also substantiated that the NBF stage was independently associated with an increased risk of CSS. In patients with non-metastatic colon cancer, a 77.1% increased risk of cancer-specific mortality was observed (HR = 1.771, 95% CI: 1.569–2.000, *p* < 0.001; Supplementary Table S1), which was higher than that in the overall cohort, indicating that the prognostic prediction efficacy of NBF stage improved in patients with AJCC stage I–III colon cancer.

### 3.3. Prognostic Prediction of NBF-TNM Stage

**Figure 4:**
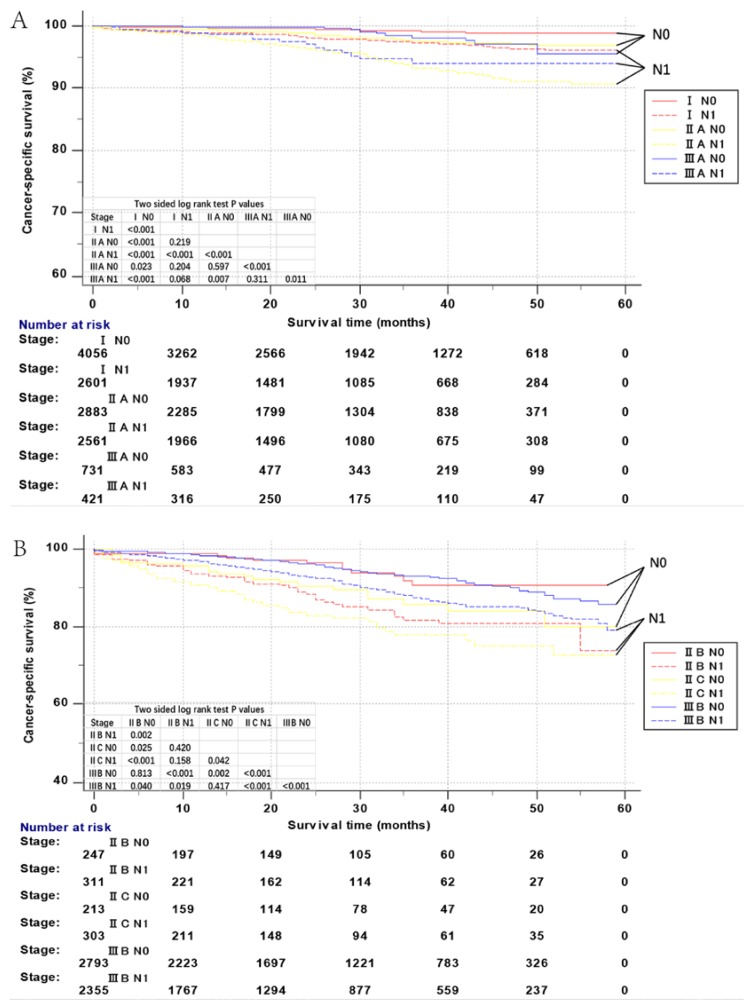

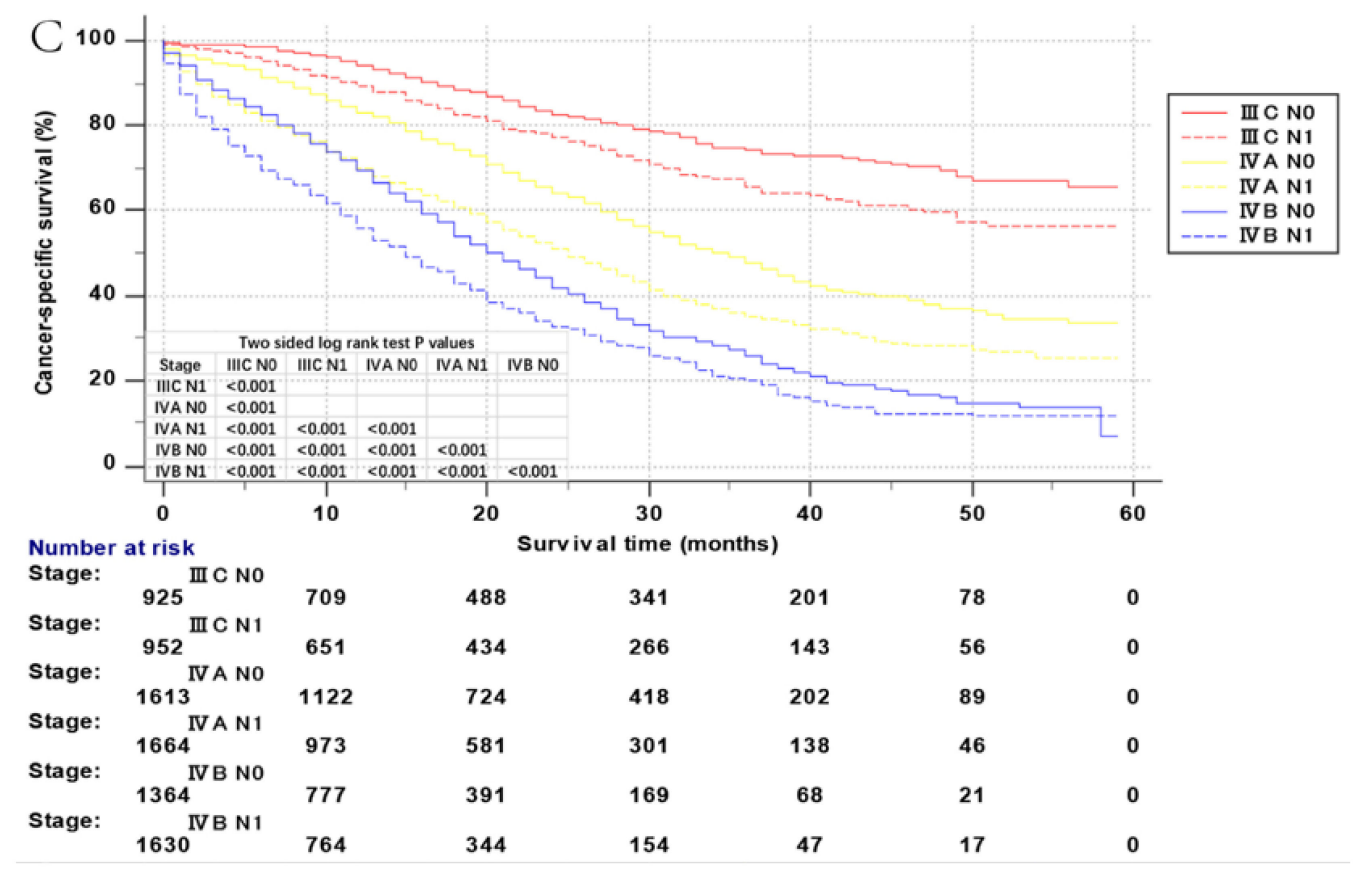
Kaplan–Meier survival curves of non-biological factor-Tumor-Node-Metastasis (NBF-TNM) staging system. (**A**) Cancer-specific survival (CSS) of I-N0 stage, I-N1 stage, IIA-N0 stage, IIA-N1 stage, IIIA-N0 stage, and IIIA-N1 stage. (**B**) CSS of IIB-N0 stage, IIB-N1 stage, IIC-N0 stage, IIC-N1 stage, IIIB-N0 stage, and IIIB-N1 stage. (**C**) CSS of IIIC-N0 stage, IIIC-N1 stage, IVA-N0 stage, IVA-N1 stage, IVB-N0 stage, and IVB-N1 stage.

The concordance index of NBF-TNM stage was 0.857 (95% CI = 0.8472–0.8668). Kaplan–Meier CSS of all NBF–TNM stages (AJCC TNM staging system including I, IIA, IIB, IIC, IIIA, IIIB, IIIC, IVA, and IVB, combined with NBF 0 or NBF1 stage) was used for the analysis of the prognostic prediction of the NBF–TNM stage in the overall cohort (*n* = 28,818), as seen in Figure 4A,C. As expected, all NBF0-stage patients showed a statistically significant increased CSS as compared to the NBF1-stage patients (*p* < 0.05) in all the respective AJCC TNM stages.

Moreover, Figure 4A,C also shows an increased or not apparently different, 59-month CSS of stage NBF0–TNM patients as compared to stage NBF1–TNM patients with higher risk AJCC stages. For example, an increased CSS was found in stage IIA-NBF0 as compared to stage IIIA-NBF1 (*p* < 0.001). Similarly, we also noted a decreased CSS in stage IIB-NBF1 as compared to stage IIIB-NBF0 (*p* < 0.001), and not apparently different CSS in stage I-NBF1 as compared to stage III-NBF0 (*p* = 0.204).

The multivariate Cox regression analyses compared the HRs of each AJCC TNM stage and NBF-TNM stages. Consistent with the Kaplan–Meier survival curves, all the NBF0-TNM patients showed lower HRs as compared to the respective NBF1-TNM stages (Table 4). Notably, several node-positive stages (stage IIIA-NBF0, IIIA-NBF1, IIIB-NBF0, or IIIB-NBF1) had a better prognosis than that of several node-negative stages (stage IIC-NBF0, IIC-NBF1, or IIB-NBF1). In addition, HRs of several stages NBF1-TNM patients even exceeded stage NBF0-TNM patients, displaying higher risk by conventional AJCC TNM stages. For example, the cancer-specific mortality was higher in stage I-NBF1 patients (HR = 3.172, 95% CI: 2.009–5.008) as compared to stage IIA-NBF0 (HR = 2.391, 95% CI: 1.503–3.801), or IIIA-NBF0 patients (HR = 2.153, 95% CI: 1.072–4.326). In stage IIIA-NBF1 patients (HR = 5.595, 95% CI: 2.987–10.478) as compared to stage IIA-NBF0. In stage IIB-NBF1 patients (HR = 18.142, 95% CI: 11.143–29.537) as compared to stage IIC-NBF0 (HR = 14.397, 95% CI: 8.217–25.224), or IIIB-NBF0 patients (HR = 8.364, 95% CI: 5.594–12.506). The above phenomena indicated that the NBF-TNM stage greatly improved the accuracy of prognostic prediction than the conventional AJCC TNM stage after combining with the NBF-stage, thereby demonstrating that the NBF1-stage exhibited an upstage effect in some patients with the TNM stage of colon cancer. Thus, the prognostic prediction efficacy was found to be robust in patients with non-metastatic colon cancer.

## 4. Discussion

Nowadays, enormous progress has been made on the cellular and molecular biology level in colon cancer [13,14]. However, only a few studies focused on the prognosis of NBFs. Furthermore, none of them analyzed more than three NBFs in one study, and none of them combined the NBFs with the existing staging system for a superior prognostic prediction and clinical management. In 2013, a large population-based study showed that married patients were at low risk to present with metastatic disease and more likely to receive effective treatment, as compared to the unmarried patients who faced a significantly higher risk of mortality with colon cancer [6]. A similar conclusion was obtained in three studies [5,9,15], and another previous study found that marriage could result in improved cardiovascular, endocrine, and immune function [16]. We also believed that the depression caused by not being married was related to Vascular Endothelial Growth Factor (VEGF), which could stimulate endothelial cell migration, proliferation and proteolytic activity [17]. Reportedly, Medicaid status or no insurance was associated with unfavorable survival [7,8,15]. We held the view that the following three reasons might lead to the poor prognosis of Medicaid status: Medicaid beneficiaries are initiating treatment late, or receiving inadequate treatment; adults enrolled in the Medicaid program are likely to be disabled, presenting with psychiatric and/or physical comorbidities; and these patients would encounter various barriers (e.g., transportation, poor psychosocial support) that may hinder receipt of adequate treatment and follow-up care [8]. Furthermore, the current results concerning the prognosis of marital status and insurance status, were in agreement with previous studies. The current analyses also showed that the higher the county-level median household income of patients in one group, the better the prognosis except in the “19.15K–48.57K” group. Furthermore, a lower median household income was found to be associated with poor survival of patients with malignant colon cancer, considering that the patients with low income have a fragile financial support network for coping with the challenges of colon cancer treatment. With regard to the non-uniform effect of income on survival, our results were consistent with a previous study in ovarian cancer [18]. We thought this strange phenomenon was mainly because of the various relief policies provided by the US government to the low-incomes.

In addition, the results of the current study also showed that the other three NBFs (county percentage with a bachelor’s degree, unemployment situation, and year of diagnosis) were not significant prognostic factors in multivariate Cox regression analysis.

The AJCC staging system is widely accepted and clinically used worldwide, although it only considers the extent of invasion of the primary tumor, number of lymph nodes, and distant spread [19], and does not consider the other biological factors that influence the prognosis of colon cancer. Although several previous modifications have improved the predictive ability of the stage, it is not yet optimal for the prediction of prognosis. In 2011, AJCC proposed additional refined staging methods based on the other available factors beyond the classic tumor node metastases (TNM) staging [10]. Consequently, the need for a comprehensive staging, combined with other biological and non-biological factors is a major concern.

However, to the best of our knowledge, the NBFs have not yet been well studied in the prognosis of colon cancer and the current study is the first to incorporate NBFs into AJCC staging system.

Herein, the new proposed NBF stage (based on marital status, insurance status, and county-level median household income) was demonstrated to be an independent prognostic factor, and all NBF1-stage patients showed significantly increased mortality as compared to the NBF0-stage patients with the same TNM stage. Furthermore, our analyses revealed that NBF1-stage had a 50.4% increased risk of cancer-specific mortality in colon cancer, which rose to 77.1% in non-metastatic colon cancer. Distinguishing between stages IIIA-NBF0 and IIIA-NBF1 in the TNM stage IIIA accounted for the improved prognosis of TNM stage IIIA than IIA [2,10].

Moreover, we also found several NBF1-TNM stages exceeded the NBF0-TNM stages with higher TNM stages. Reportedly, a better prognosis was noted in TNM stage I than stage IIIA, in TNM stage IIIA than stage IIA, in TNM stage IIB than stage IIIB, and in TNM stage IIIB than stage IIC [10]. However, the current analysis showed that the cancer-specific mortality was higher in stage I-NBF1 patients as compared to stage IIA-NBF0 or IIIA-NBF0 patients, in stage IIIA-NBF1 patients as compared to stage IIA-NBF0, in stage IIB-NBF1 patients as compared to stage IIC-NBF0, or IIIB-NBF0 patients. Thus, the NBF stage should be incorporated into the conventional AJCC TNM staging system, which is primarily based on several disease-related biological factors. NBF-TNM stage would improve the prognostic prediction in colon cancer, especially non-metastatic colon cancer.

The superior prognosis of several node-positive stages (stage IIIA-NBF0, IIIA-NBF1, IIIB-NBF0, or IIIB-NBF1) than that of several node-negative stages (stage IIC-NBF0, IIC-NBF1, or IIB-NBF1) ascribed a drawback of the AJCC TNM staging system: Some node-negative patients exhibited poor prognosis, and not all patients with node-positive status were associated with poor prognosis [20,21]. In addition, the stage I (T1–T2N0M0)–NBF1-stage had a higher HR than stage IIIA (T1–T2N1M0)–NBF0. Considering almost the same in the T-stage (T1–T2), we suspected that the NBF-1 might be more robust than the node-positive status for indicating a poor prognosis. However, in the clinical treatment, stage IIIA, not stage I, patients are treated with adjuvant chemotherapy [22]. Therefore, the current study suggested the existence of undertreatment in the TNM stage I colon cancer, and overtreatment in the TNM stage IIIA colon cancer. In addition, NBF1-stage could play a role in guiding the application of chemotherapy considering the role of node-positive status in the application of chemotherapy [23,24]. The phenomenon that stage I (T1–T2N0M0)–NBF1 had higher HR than stage IIA (T3N0M0)–NBF0, and stage IIB (T4aN0M0)–NBF1 had higher HR than stage IIC (T4bN0M0)–NBF0, showed that NBF1-stage might be stronger than T3 and T4b stages for indicating a poor prognosis.

Chemotherapy is a critical adjuvant therapy for colon cancer and has been studied extensively in the past decades. Nowadays, it has been widely accepted that TNM stage II with any of the high-risk factors (T4-stage, obstruction, perforation, poorly differentiated histology, <12 lymph nodes, the presence of lymphovascular or perineural invasion, or positive margins) should be considered to receive adjuvant chemotherapy [25,26,27,28,29]. However, some researchers reported that patients with stage II colon cancer with any high-risk factors did not exhibit substantial survival benefit from adjuvant chemotherapy [26,30]. Consequently, we proposed that NBF-stage might improve this situation, and some patients with stage II colon cancer with one or some high-risk factors should be spared from adjuvant chemotherapy.

Nevertheless, the present study had several limitations. First, the NBF–TNM stage did not consider other biological prognostic factors, including microsatellite instability status, treatment, and CEA (carcinoembryonic antigen) level, which might affect survival [22,31,32]; thus, NBF–TNM stage necessitates further refinement in future studies. Second, limited by the SEER database, the sample size is relatively small and needs to be enlarged. The longest follow-up time was only 59 months and did not exceed 5 years. Furthermore, our study was based on a US-population, and our results might not apply to other countries. For example, as far as we knew, most of the European health systems allowing patients free access to cancer services might greatly reduce the effect of insurance status on survival. Finally, considering the analyses were based merely on retrospective data, prospective clinical studies with respect to NBF-stage were essential for an accurate prediction of prognosis and improvement in clinical management.

## 5. Conclusions

In conclusion, the current study demonstrated that marital status, insurance status, and median household income were significant prognostic factors in colon cancer, while NBF-stage was an independent prognostic factor. Thus, NBFs that are otherwise neglected in clinical practice necessitate intensive focus in future studies. Furthermore, healthcare professionals and institutions in charge of patients with colon cancer, should pay more attention to rectal cancer patients with poor NBFs who may benefit from additional resources and support during their therapy. Taken together, the improved precision of prognostic prediction and the guidance of adjuvant chemotherapy in TNM stage II and stage III colon cancer, strongly support the incorporation of NBF-stage into conventional AJCC TNM staging system.

## Figures and Tables

**Table 1:** Baseline characteristics of colon cancer patients included in our study.

Characteristic		No. (%)
Race	White	21,179 (73.5)
	Black	4706 (16.3)
	Other	2933 (10.2)
Gender	Male	15,402 (53.4)
	Female	13,416 (46.6)
Tumor location	Appendix	359 (1.2)
	Cecum	5276 (18.3)
	Ascending colon	4703 (16.3)
	Hepatic flexure	1165 (4.0)
	Transverse colon	2538 (8.8)
	Splenic flexure	1062 (3.7)
	Descending colon	2350 (8.2)
	Sigmoid Colon	11,365 (39.4)
Tumor grade	Grade I	2360 (8.2)
	Grade II	19,568 (67.9)
	Grade III	4103 (14.2)
	Grade IV	723 (2.5)
	Unknown	2064 (7.2)
AJCC stage	I	6926 (24.0)
	IIA	5645 (19.6)
	IIB	589 (2.0)
	IIC	546 (1.9)
	IIIA	1182 (4.1)
	IIIB	5323 (18.5)
	IIIC	1938 (6.7)
	IVA	3461 (12.0)
	IVB	3208 (11.1)
Surgery	Surgery performed	26,296(91.2)
	Surgery not performed	2522(8.8)
County % with bachelor degree	5.95–20.77%	7070 (24.5)
	20.78–29.91%	5826 (20.2)
	29.92–35.57%	8689 (30.2)
	35.58–64.01%	7233 (25.1)
County-level median household income ^#^	19.15–48.57 K	7180 (24.9)
	48.58–55.87 K	6719 (23.3)
	55.88–67.29 K	7587 (26.3)
	67.30–106.52 K	7332 (25.4)
County % were unemployed	1.92–8.66%	7180 (24.9)
	8.67–9.60%	6719 (23.3)
	9.61–11.27%	7587 (26.3)
	11.28–21.21%	7332 (25.4)
Year of diagnosis	2010	5757 (20.0)
	2011	5634 (19.6)
	2012	5596 (19.4)
	2013	5773 (20.0)
	2014	6058 (21.0)
Tumor size	≤5 cm	16,409 (56.9)
	>5 cm	8409 (29.2)
	Unknown	4000 (13.9)
Age at diagnosis (years)	≤50	8245 (28.6)
	51–55	6313 (21.9)
	56–60	7335 (25.5)
	≥61	6925 (24.0)
Insurance status	Insured	21,198 (73.6)
	Medicaid	5258 (18.2)
	Uninsured	2362 (8.2)
Marital status	Married	17,515 (60.8)
	Single	7040 (24.4)
	Divorced	3321 (11.5)
	Widowed	942 (3.3)

^#^ Shown in US dollars.

**Table 2:** Multivariate Cox regression analyses of CSS.

Variable	Reference	Characteristic	Cancer-Specific Survival		
			HR (95%)	SE	*p* Value
Race	White	Black	1.179 (1.089–1.275)	0.040	<0.001
		Other	1.046 (0.941–1.162)	0.054	0.405
Gender	Male	Female	0.851 (0.801–0.904)	0.031	<0.001
Tumor location	Appendix	Cecum	0.871 (0.682–1.113)	0.125	0.270
		Ascending colon	0.864 (0.675–1.107)	0.126	0.249
		Hepatic flexure	0.859 (0.652–1.132)	0.141	0.281
		Transverse colon	0.811 (0.626–1.050)	0.132	0.112
		Splenic flexure	0.773 (0.585–1.023)	0.143	0.072
		Descending colon	0.670 (0.516–0.869)	0.133	0.003
		Sigmoid Colon	0.620 (0.487–0.791)	0.124	<0.001
Tumor grade	Grade I	Grade II	1.042 (0.892–1.217)	0.079	0.601
		Grade III	1.720 (1.461–2.025)	0.083	<0.001
		Grade IV	2.111 (1.716–2.596)	0.106	<0.001
		Unknown	1.164 (0.980–1.382)	0.088	0.083
AJCC stage	I	IIA	2.266 (1.738–2.955)	0.135	<0.001
		IIB	6.547 (4.614–9.291)	0.179	<0.001
		IIC	9.819 (7.124–13.535)	0.164	<0.001
		IIIA	1.864 (1.199–2.899)	0.225	0.006
		IIIB	5.600 (4.399–7.129)	0.123	<0.001
		IIIC	16.630 (13.051–21.192)	0.124	<0.001
		IVA	39.280 (31.324–49.257)	0.115	<0.001
		IVB	54.228 (43.145–68.159)	0.117	<0.001
Surgery	Surgery performed	Surgery not performed	2.649 (2.417–2.903)	0.047	<0.001
Insurance status	Insured	Medicaid	1.482 (1.376–1.597)	0.038	<0.001
		Uninsured	1.620 (1.476–1.778)	0.048	<0.001
Marital status	Married	Single	1.207 (1.123–1.297)	0.037	<0.001
		Divorced	1.064 (0.969–1.169)	0.048	0.195
		Widowed	1.114 (0.945–1.314)	0.084	0.199
Tumor size	≤5 cm	>5 cm	1.157 (1.080–1.240)	0.035	<0.001
		Unknown	1.187 (1.082–1.302)	0.047	<0.001
Age at diagnosis (years)	≤50	51–55	1.078 (0.988–1.175)	0.044	0.092
		56–60	1.188 (1.096–1.288)	0.041	<0.001
		≥61	1.338 (1.232–1.452)	0.042	<0.001
County % with bachelor degree	35.58–64.01%	29.92–35.57%	1.017 (0.918–1.127)	0.052	0.745
		20.78–29.91%	1.035 (0.926–1.157)	0.057	0.547
		5.95–20.77%	1.138 (1.005–1.289)	0.063	0.041
County-level median household income	67.30–106.52 K	55.88–67.29 K	1.150 (1.037–1.276)	0.053	0.008
		48.58–55.87 K	1.154 (1.027–1.297)	0.060	0.016
		19.15–48.57 K	1.132 (1.001–1.281)	0.063	0.048
County % were unemployed	1.92–8.66%	8.67–9.60%	1.074 (0.984–1.171)	0.044	0.110
		9.61–11.27%	1.014 (0.922–1.115)	0.049	0.771
		11.28–21.21%	1.062 (0.968–1.265)	0.047	0.201

**Table 3:** Multivariable Cox regression analyses of independent prognostic factors in colon cancer.

Variable	Reference	Characteristic	Cancer-Specific Survival		
			HR (95%)	SE	*p* Value
Race	White	Black	1.210 (1.121–1.307)	0.039	<0.001
		Other	1.038 (0.936–1.152)	0.053	0.477
Gender	Male	Female	0.842 (0.793–0.894)	0.031	<0.001
Tumor location	Appendix	Cecum	0.897 (0.702–1.146)	0.125	0.386
		Ascending colon	1.881 (0.688–1.129)	0.126	0.317
		Hepatic flexure	0.878 (0.667–1.156)	0.140	0.353
		Transverse colon	0.824 (0.637–1.067)	0.132	0.143
		Splenic flexure	0.785 (0.594–1.038)	0.143	0.09
		Descending colon	0.680 (0.524–0.882)	0.133	0.004
		Sigmoid Colon	0.637 (0.500–0.811)	0.124	<0.001
Tumor grade	Grade I;	Grade II	1.027 (0.879–1.199)	0.079	0.737
		Grade III	1.698 (1.443–1.999)	0.083	<0.001
		Grade IV	2.076 (1.688–2.554)	0.106	<0.001
		Unknown	1.152 (0.970–1.368)	0.088	0.106
Surgery	Surgery performed	Surgery not performed	2.632 (2.402–2.884)	0.047	<0.001
Tumor size	≤5 cm	>5 cm	1.169 (1.090–1.252)	0.035	<0.001
		Unknown	1.186 (1.081–1.300)	0.047	<0.001
Age at diagnosis (years)	≤50	51–55	1.086 (0.995–1.184)	0.044	0.063
		56–60	1.189 (1.097–1.289)	0.041	<0.001
		≥61	1.340 (1.236–1.454)	0.041	<0.001
County % with bachelor degree	5.95–20.77%	20.78–29.91%	1.072 (0.978–1.176)	0.047	0.136
		29.92–35.57%	1.119 (1.021–1.228)	0.047	0.017
		35.58–64.01%	1.254 (1.139–1.382)	0.049	<0.001
County % were unemployed	1.92–8.66%	8.67–9.60%	1.090 (1.000–1.188)	0.044	0.050
		9.61–11.27%	1.050 (0.959–1.150)	0.047	0.293
		11.28–21.21%	1.075 (0.981–1.178)	0.047	0.121
AJCC stage	I	IIA	2.296 (1.761–2.994)	0.135	<0.001
		IIB	6.704 (4.725–9.512)	0.178	<0.001
		IIC	9.979 (7.240–13.753)	0.164	<0.001
		IIIA	1.859 (1.195–2.890)	0.225	0.006
		IIIB	5.651 (4.439–7.194)	0.123	<0.001
		IIIC	16.921 (13.279–21.562)	0.124	<0.001
		IVA	40.051 (31.941–50.220)	0.115	<0.001
		IVB	55.404 (44.081–69.636)	0.117	<0.001
NBF stage	Stage 0	Stage 1	1.504 (1.415–1.600)	0.031	<0.001

**Table 4:** Prognosis of NBF-TNM stage in colon cancer.

Stage	No. of Patients	Cancer-Specific Survival		
		HR (95% CI)	SE	*p* Value
**I NBF0**	4190	1.000 (Referent)
**I NBF1**	2736	3.172 (2.009–5.008)	0.233	*p* < 0.001
**IIA NBF0**	2975	2.391 (1.503–3.801)	0.237	*p* < 0.001
**IIA NBF1**	2670	6.570 (4.348–9.928)	0.211	*p* < 0.001
**IIB NBF0**	258	7.130 (3.689–13.781)	0.336	*p* < 0.001
**IIB NBF1**	331	18.142 (11.143–29.537)	0.249	*p* < 0.001
**IIC NBF0**	223	14.397 (8.217–25.224)	0.286	*p* < 0.001
**IIC NBF1**	323	23.679 (14.859–37.736)	0.238	*p* < 0.001
**IIIA NBF0**	748	2.153 (1.072–4.326)	0.356	*p* < 0.001
**IIIA NBF1**	434	5.595 (2.987–10.478)	0.320	*p* < 0.001
**IIIB NBF0**	2867	8.364 (5.594–12.506)	0.205	*p* < 0.001
**IIIB NBF1**	2456	13.382 (9.005–19.886)	0.202	*p* < 0.001
**IIIC NBF0**	949	26.248 (17.553–39.249)	0.205	*p* < 0.001
**IIIC NBF1**	989	38.634 (25.996–57.416)	0.202	*p* < 0.001
**IVA NBF0**	1681	61.161 (41.193–89.505)	0.194	*p* < 0.001
**IVA NBF1**	1780	92.326 (63.182–134.911)	0.194	*p* < 0.001
**IVB NBF0**	1435	91.451 (62.434–133.954)	0.195	*p* < 0.001
**IVB NBF1**	1773	121.179 (82.852–177.236)	0.194	*p* < 0.001

## Supplementary Materials

The following are available online at http://www.mdpi.com/2072-6694/10/8/263/s1, Table S1: Multivariable Cox regression analyses of independent prognostic factors in non-metastatic colon cancer.
