# Supplementary material for: Real-World Implications of Nonbiological Factors with Staging, Prognosis and Clinical Management in Colon Cancer
Source: Cancers (Basel). 2018 Aug 8;10(8):263. doi: 10.3390/cancers10080263 (PMC6115817; doi:10.3390/cancers10080263)
Supplement: Supplementary file 1 [file cancers-10-00263-s001.pdf]

Supplementary Materials: Real-World Implications of Nonbiological Factors with Staging, Prognosis and Clinical Management in Colon Cancer

Qi Liu, Dakui Luo, Sanjun Cai, Qingguo Li and Xinxiang Li

Table S1. Multivariable Cox regression analyses of independent prognostic factors in non–metastatic colon cancer. (Abbreviations: HR, hazard ratio; AJCC, American Joint Committee on Cancer; NBF, non-biological factor)

Variable	Reference	Characteristic	Cancer-Specific Survival		
			HR (95%)	SE	*p*-value
**Race**	White	Black	1.354 (1.168–1.570)	0.075	<0.001
		Other	0.985 (0.802–1.208)	0.104	0.881
**Gender**	Male	Female	0.817 (0.727–0.919)	0.06	0.001
**Tumor location**	Appendix	Cecum	0.631 (0.408–0.976)	0.222	0.038
		Ascending colon	0.556 (0.357–0.866)	0.226	0.009
		Hepatic flexure	0.624 (0.381–1.022)	0.251	0.061
		Transverse colon	0.579 (0.365–0.918)	0.235	0.020
		Splenic flexure	0.686 (0.420–1.120)	0.250	0.132
		Descending colon	0.502 (0.315–0.802)	0.239	0.004
		Sigmoid Colon	0.444 (0.289–0.683)	0.220	<0.001
**Tumor grade**	Grade Ⅰ	Grade Ⅱ	1.139 (0.867–1.496)	0.139	0.351
		Grade Ⅲ	1.878 (1.405–2.511)	0.148	<0.001
		Grade Ⅳ	2.284 (1.572–3.320)	0.191	<0.001
		Unknown	1.189 (0.779–1.814)	0.216	0.422
**Surgery**	Surgery performed	Surgery not performed	9.343 (6.984–12.500)	0.149	<0.001
**Tumor size**	≤5 cm	>5 cm	1.022 (0.900–1.161)	0.065	0.735
		Unknown	1.282 (1.011–1.627)	0.122	0.041
**Age at diagnosis (years)**	≤50	51–55	1.033 (0.868–1.231)	0.089	0.712
		56–60	1.387 (1.184–1.624)	0.081	<0.001
		≥61	1.458 (1.243–1.711)	0.082	<0.001
**County % with bachelor degree**	5.95%–20.77%	20.78%–29.91%	1.218 (1.015–1.462)	0.093	0.034
		29.92%–35.57%	1.164 (0.968–1.400)	0.094	0.106
		35.58%–64.01%	1.366 (1.126–1.656)	0.098	0.002
**County % were unemployed**	1.92%–8.66%	8.67%–9.60%	1.095 (0.921–1.301)	0.088	0.303
		9.61%–11.27%	1.051 (0.877–1.259)	0.092	0.591
		11.28%–21.21%	1.148 (0.960–1.373)	0.091	0.129
**AJCC stage**	I	ⅡA	2.557 (1.942–3.367)	0.140	<0.001
		ⅡB	7.243 (5.059–10.369)	0.183	<0.001
		ⅡC	9.094 (6.520–12.684)	0.170	<0.001
		ⅢA	2.003 (1.286–3.112)	0.226	0.002

		ⅢB	6.341 (4.932–8.154)	0.128	<0.001
		ⅢC	19.014 (14.722–24.557)	0.131	<0.001
**NBF stage**	Stage 0	Stage 1	1.771 (1.569–2.000)	0.062	<0.001

© 2018 by the authors. Licensee MDPI, Basel, Switzerland. This article is an open access article distributed under the terms and conditions of the Creative Commons Attribution (CC BY) license (http://creativecommons.org/licenses/by/4.0/).
